# Empowerment and health care access barriers among currently married women in Myanmar

**DOI:** 10.1186/s12889-021-10181-5

**Published:** 2021-01-15

**Authors:** Nang Mie Mie Htun, Zar Lwin Hnin, Win Khaing

**Affiliations:** Department of Preventive and Social Medicine, University of Medicine Mandalay, Ministry of Health and Sports, Mandalay, 100102 Myanmar

**Keywords:** Women’s empowerment, Barriers to accessing health care, Myanmar

## Abstract

**Background:**

Although Myanmar is moving to attain UHC in 2030, health care utilization indicators are still low, especially among women. Women’s health outcomes are determined by the lack of access to health care, and many factors influence this condition. The objective of the present work was to identify the association between women’s empowerment and barriers to accessing health care among currently married women in Myanmar.

**Method:**

We performed a secondary analysis using the Myanmar Demographic and Health Survey (2015–16), including 7759 currently married women aged 15–49 years. The outcome variable, barriers to accessing health care, were asked about in terms of whether the respondent faced barriers to getting permission to go, getting money to go, the distance to the health facility, and not wanting to go alone. The variables were recoded into zero, one, and more than one barrier. After performing the exploratory factor analysis for women’s empowerment indicators (decision-making power and disagreement to justification to wife-beating), a multinomial logistic regression was carried out.

**Results:**

Among currently married women, 48% experienced no barriers when accessing health care services, 21.9% had one barrier, and 30.1% had more than one barrier. After the exploratory factor analysis, scores were recoded into three levels. Women with low and middle empowerment had 1.5 odds (AOR 1.5, 95% CI: 1.2–1.8) and 1.5 odds (AOR 1.5, 95% CI: 1.3–1.9), respectively, to have barriers to accessing health care when compared to those with high empowerment for one barrier group. For the women who had more than one barrier, women with low empowerment were 1.4 times more likely (AOR 1.4, 95% CI: 1.1–1.7) to experience barriers in comparison to women with high empowerment. The barriers were seen to be reduced in the case of women who had a high level of education, had fewer children, came from rich households, and lived in urban areas.

**Conclusion:**

When women are more empowered, they tend to face fewer barriers when accessing health care services. This finding could contribute to the policy formulation for reducing health inequity issues by increasing women’s empowerment.

**Supplementary Information:**

The online version contains supplementary material available at 10.1186/s12889-021-10181-5.

## Background

All United Nations Member States have agreed to try to achieve universal health coverage (UHC) by 2030 as part of the Sustainable Development Goals. Many countries are already making progress towards UHC, but a lot of people are still left behind, especially vulnerable groups, including women and children from developing countries. Access to all-inclusive and high-quality health care is essential for promoting and maintaining health, preventing and managing diseases, reducing premature deaths, and achieving health equity for all.

Myanmar is one of the countries moving towards Universal Health Coverage in 2030. Though out-of-pocket financing has decreased from 81 to 65% of Myanmar’s total health expenditure between 2014 and 2015 after increases in public spending, this kind of financing remains the primary method of payment for health services in the country. Though the government of Myanmar aims to extend access to a Basic Essential Package of Health Services (EPHS) to the entire population by 2020, as of the time of data collection, health facilities charged patients fees for maternal and child health services [1, 2]. In 2018, a study stated that the attainment of universal health coverage in Myanmar in the immediate future would be very challenging as a result of the low health service coverage, high financial risk, and inequalities in access to healthcare. Health care utilization indicators are low, especially for women. The rates of such indicators are low (family planning needs satisfied: 75.9%; at least four antenatal care visits: 55.5%; full immunization: 55.2%; institutional delivery: 37.1%; skill birth attendance: 60.2%) [3].

In recent years, women’s empowerment has become an important global issue. The term ‘women’s empowerment’ can be defined as the ability of women to make their own decisions and act accordingly [4, 5]. Women’s empowerment is context-specific, and it is determined by various factors. Women’s empowerment is influenced by a woman’s level of education, employment for cash status, extent of media exposure, and spousal age difference [6]. There is substantial evidence that the lives of women living in low-income countries are characterized by exclusion, and this is reflected in their poor access to basic health care and services [7]. Women’s empowerment has a profound influence on the use of health services that could be linked to reproductive health outcomes [8, 9]. Women’s empowerment can control the household’s decisions regarding health care usage. In many areas—especially rural areas—men often control decisions about the health of their wives and children, including the family’s use of health services [4, 10]. A group of studies that were mainly conducted in Asia and Africa showed that women’s empowerment is linked with contraception usage [11, 12], lower fertility [13], and longer birth intervals [14].

According to the Global Gender Gap Report 2020, the global gender gap index rank for Myanmar is 114 out of 153 countries. Among four indicators (politics, economic, education, and health), the health indicator gap is the smallest [15]. The main indicators leading to the big gender gap are politics and economic, but there are many inconsistencies in the case of health indicators. In the recent Myanmar census report (2014), the maternal mortality ratio was 282 per 100,000 live births, the second-highest among Southeast Asian countries [16]. The majority (62%) of maternal deaths occurred at home, and 14% occurred on the way to the hospital due to late referrals, primary delays, and long travel distances [17]. A qualitative study was conducted in 2013on an internally displaced person who stayed in the camps in Kachin State by the Gender Equality Network. The findings of the study provided insights into the health problems experienced by women. Furthermore, a lack of access to health care by the women and inability to make their own decisions on contraceptive usage led to increased reproductive health problems and highlighted gender inequality issues in Kachin State, Myanmar [18].

There is a limited understanding in Myanmar regarding the relationship between the empowerment of women and health service utilization by married women, including reproductive, maternal, and child health services.

The present study aimed to provide an insight into the link between women’s empowerment and barriers to accessing health care services for women and potentially to inform policy around both, using a standardized index of women’s empowerment among currently married women by using Myanmar Demographic and Health Survey (MDHS) (2015–16) data.

## Methods

This study used data from the first MDHS, which was conducted between 2015 and 2016. The DHS was a nationally representative cross-sectional survey on demographic and health indicators of women and members of their households and was implemented by the Ministry of Health and Sports, Myanmar, with technical assistance from the ICF (Inner City Fund) (Rockville, Maryland, USA). Detailed methods and data collection procedures have been published elsewhere [19]. Briefly, a two-stage cluster sampling design (441 clusters, 30 households per cluster) was used and stratified by urban and rural status in 15 states and regions. Administratively, Myanmar consists of seven states representing the mountainous areas and eight regions representing the plain area; most of the regions are relatively developed [16]. Rural and urban areas are defined according to the Ministry of Home Affairs, Myanmar. According to the ministry’s definition, to be categorized as ‘urban,’ an area should meet more than 20 criteria (e.g., a large population and the availability of basic public services such as transportation, electricity, and safe drinking water) [20].

**Fig. 1:**
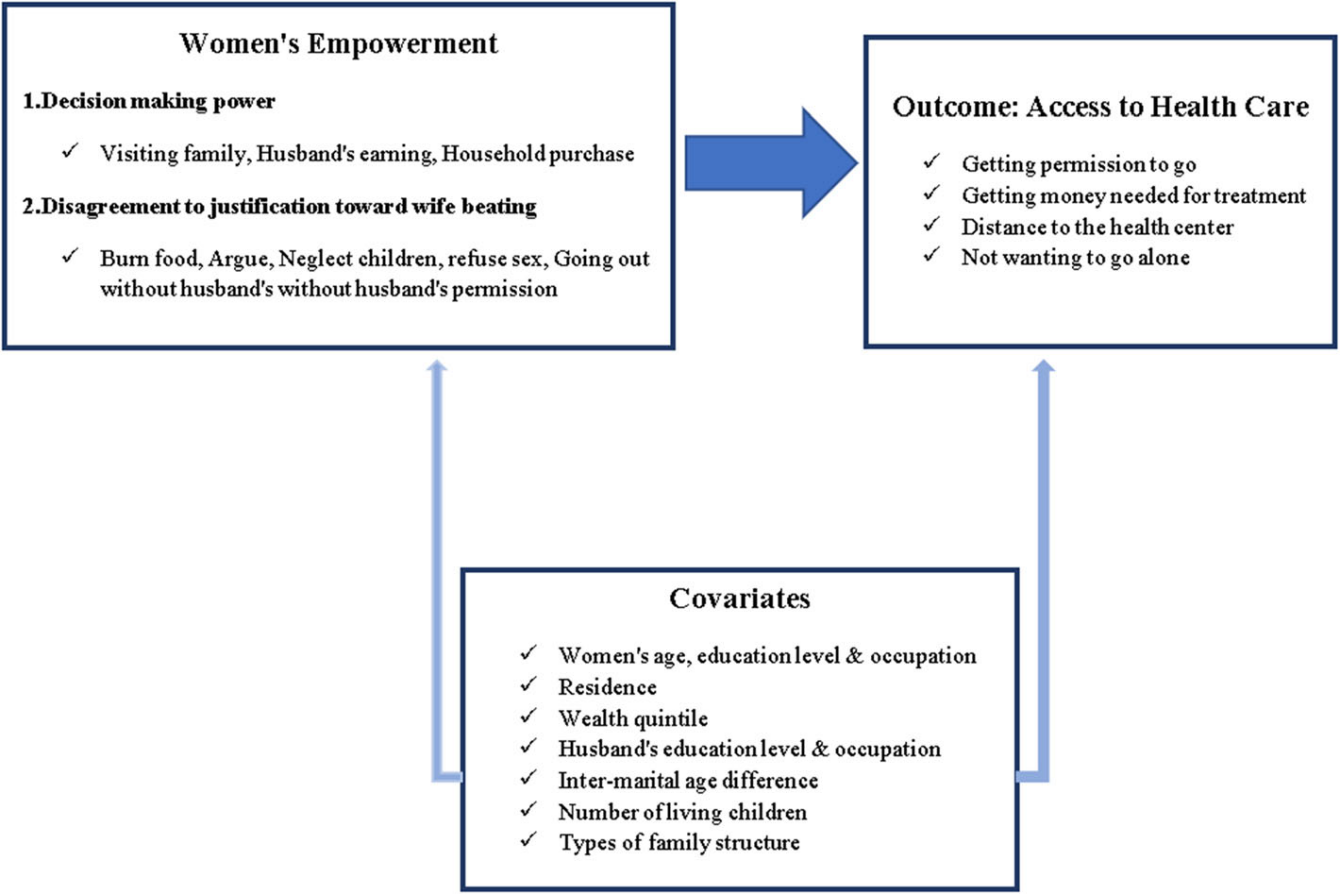
Conceptual framework

A standardized questionnaire was used to collect the data on demographic, social, and behavioural indicators, including the health status and reproductive health of all men and women aged between 15 and 49 years in the selected households. The focus of the analysis was on 7759 eligible currently married women aged 15–49 years. The sample was restricted to married women because some of the indicators used to calculate women’s empowerment are applicable only to currently married women—most notably, decision-making power was not applicable to unmarried women. A conceptual framework was constructed to meet the aim of the study (Fig. 1).

### Exposure variables

According to the Guide to DHS statistics DHS-7 version 2, 8 variables were selected as women’s empowerment indicators [21]. They can be categorized into two domains—namely, decision-making power and women’s disagreement with the justification of wife-beating [6–12]. Decision-making power was assessed through three items household purchases, visits to family members, and husband’s earnings. Women’s disagreement with the justification of wife-beating was evaluated based on five items: neglecting children, going out without husband’s permission, arguing with husband, refusing sex, and burning food. Exploratory factor analysis was performed by analyzing eight variables to extract the main factor components. Sampling adequacy and inter-correlation of variables were checked using Bartlett’s test of sphericity and Kaiser-Meyer-Olkin’s measure (0.78). The number of components was determined based on the Kaiser criterion (eigenvalues > 1) and scree plots. For ease of interpreting the factors, oblique rotation was performed. The main factor component was obtained, which was mainly contained of women’s disagreement with the justification of wife-beating regarding neglecting children and going out without their husband’s permission. These disagreements accounted for 89% of the total variance (See Additional file 1). The factor score was categorized into terciles of low, medium, and high levels of the women’s empowerment indicator.

### Outcome variable

Barriers to accessing health care were the outcome variables of interest. In the MDHS 2015–16, it was defined the percentage of currently married women age 15–49 who reported that they have experienced serious barriers to accessing health care for themselves when they are sick. Responses were categorized by type of barrier: (1) Getting permission to go to the doctor? (2) Getting money needed for advice or treatment? (3) The distance to the health facility? (4) Not wanting to go alone? [21]. All four indicators were pooled together as a single entity and recoded into three groups (0 = no barriers at all, 1 = had faced one barrier, 2 = had faced more than one barrier).

Other relevant socio-demographic factors included in the MDHS data were considered as independent factors in the analysis. The variable of husband’s occupation was recorded as either white-collar (professional/technical/managerial/clerical) or blue-collar (agricultural/manual). DHS sample weights were used in all analyses to make the sample data representative of the entire population [19]. Before doing the multinomial logistic regression analyses with confounders adjusted for the survey sampling design, the multicollinearity between variables was checked, and univariate analysis was done. Only variables with significant associations (*p*-value < 1) in the univariate analyses were included in the multinomial logistic regression. Reference categories were set according to the MDHS (2015–16) report [13] and a specific coding system [20]. All analyses used the svyset command in STATA 14.

## Results

### Background characteristics

Table 1 shows the background characteristics of currently married women aged 15–49 years. Among them, most of the women were aged between 20 and 39. About 74% were living in rural areas. Nearly half of the women were from the poorer and poorest households. Most of the women were working and had primary education. In terms of familial and marital composition, half of the women were in a nuclear family type, while 49.2% were in an extended family type. Among married couples, the majority of the wives were younger than their husbands. The education level of respondents’ husbands showed that 15.1% had no education. Only 6.7% had received higher education. Regarding the occupations of respondents’ husbands, most were blue-collar workers.

**Table 1:** Distribution of background characteristics and status of barriers in health care access among currently married women age 15–49 year

Background characteristics	Total = 7759	Barriers in health care access			Chi-square	*p*-value
		No barrier **3780 (48.0%)**	1 barrier **1725 (21.9%)**	> 1 barrier **2364 (30.1%)**		
**Women’s empowerment**						
Low	2278(33.4)	1054 (46.3)	494(21.7)	729(32.0)	70.9	< 0.001
Medium	2928(42.9)	1532(51.6)	704(23.7)	734(24.7)		
High	1620(23.7)	848(57.3)	250(16.9)	383(25.8)		
**Age**						
15–19	227(2.9)	104(46.0)	46(20.0)	77(34.0)	12.9	0.17
20–29	2092(26.7)	1016(48.5)	491(23.5)	585(28.0)		
30–39	2988(38.8)	1526(51.1)	629(21.0)	832(27.9)		
40–49	2452(31.6)	1274(51.9)	495(20.2)	685(27.9)		
**Residence**						
Urban	2022(26.1)	1256 (62.1)	461 (22.8)	305 (15.1)	242.8	< 0.001
Rural	5737(73.9)	2665 (46.5)	1200(20.9)	1872 (32.6)		
**Education**						
None	1193(15.4)	433(36.3)	231(19.4)	529(44.3)	485.9	< 0.001
Primary	3656(47.1)	1681(46.0)	811(22.3)	518(22.7)		
Secondary	2285(29.4)	1329(58.2)	518(22.7)	438(19.1)		
Higher	625(8.1)	477(76.7)	101(16.1)	45(7.2)		
**Occupation**						
Not working	2280(29.4)	1178(51.7)	514(22.5)	58(25.8)	8.9	0.09
Working	5479(70.6)	2745(50.1)	1144(20.9)	1590(29.0)		
**Wealth Index**						
Poorest	1622(20.9)	463(28.5)	342(21.1)	817(50.4)	974.2	< 0.001
Poorer	1586(20.4)	644(40.6)	376(23.7)	566(35.7)		
Middle	1555(20.1)	811(52.1)	342(21.9)	402(26.0)		
Richer	1509(19.5)	917(60.8)	331(21.9)	261(17.3)		
Richest	1487(19.1)	1086(73.0)	270(18.2)	131(8.8)		
**Husband’s occupation**						
White collar	715(9.2)	486(68.0)	130 (18.3)	99 (13.7)	101.0	< 0.001
Blue collar	7044 (90.8)	3459 (49.1)	1507(21.4)	2078 (29.5)		
**Husband’s education**						
None	1173(15.1)	467(39.8)	202 (17.2)	504 (43.0)	403.9	< 0.001
Primary	3128(40.3)	1395 (44.6)	723 (23.1)	1010 (32.3)		
Secondary	2941(37.9)	1662(56.5)	662 (22.5)	617(21.0)		
Higher	517(6.7)	402 (77.6)	77 (14.9)	38 (7.5)		
**No. of living children**						
No child	916(11.8)	496(54.2)	204 (22.2)	217 (23.6)	82.5	< 0.001
1–3	5437(70.1)	2825(52.0)	1178 (21.6)	1433 (26.4)		
4 and more	1406(18.2)	699 (42.6)	279 (19.8)	528 (37.6)		
**Inter marital age difference**						
Same age	818 (10.5)	430(52.6)	177 (21.7)	211(25.8)	7.3	0.27
Husband>wife	5274(68.0)	2616 (49.6)	1139 (21.6)	1519 (28.8)		
Wife>husband	1667(21.5)	877 (52.6)	343 (20.6)	447 (26.8)		
**Family structure**						
Nuclear	3942(50.8)	1812(46.0)	868 (22.0)	1263 (32.0)	79.9	< 0.001
Extended	3817 (49.2)	2186 (55.2)	791(20.8)	911 (24.0)		

### Barriers to health care access

In Table 1, the distribution of barriers to accessing health care among currently married women across background characteristics is shown. Among currently married women, 48.0% had no barrier while accessing health care, whereas 21.9% had one barrier, and about 30.1% had more than one barrier. Experience of barriers to accessing health care varied by respondent’s age, residence, education, occupation, wealth index, husband’s education and occupation, number of living children, inter-marital age differences, and family structure.

### Associations between women’s empowerment and barriers to health care access

Table 2 presents the multinomial regression results from the examination of the relationship between women’s empowerment and barriers to accessing health care among currently married women. We adjusted residence, education, occupation, wealth index, husband’s education and occupation, number of children, and family structure, which were significant at univariate analysis (*p*-value < 1). Women with low and middle levels of empowerment had 1.5 odds (AOR 1.5, 95% CI: 1.2–1.8) and 1.5 odds (AOR 1.5, 95% CI: 1.3–1.9), respectively, of facing barriers to accessing health care in comparison to those with high empowerment for one barrier group. Women with low empowerment were 1.4 times more likely (AOR 1.4, 95% CI: 1.1–1.7) to face more than one barrier in comparison to women with high empowerment.

**Table 2:** Association between women’s empowerment and barriers in health care access adjusted for covariates

	No barrier (***n*** = 3780) vs 1 barrier (***n*** = 1725)		No barrier (***n*** = 3780) vs > 1 barrier (***n*** = 2364)	
	OR (95% CI)	AOR (95% CI)	OR (95% CI)	AOR (95% CI)
**Women empowerment**				
Low	1.6 (1.3–2) **	1.5 (1.2–1.8) **	1.5 (1.3–1.8) **	1.4 (1.1–1.7) **
Middle	1.6 (1.3–1.9) **	1.5 (1.3–1.9) **	1.0 (0.9–1.3)	1.1 (0.9–1.3)
High	**1**	**1**	**1**	**1**
**Residence**				
Urban	**1**	**1**	**1**	**1**
Rural	1.2 (1.0–1.5) *	1.3 (1.0–1.8) *	2.9 (2.2–3.7) **	1.8 (1.6–2.1) *
**Education**				
None	2.6 (1.7–3.8) **	1.8 (1.0–2.4) *	12.9 (7.8–21.5) **	2.4 (1.4–3.9) **
Primary	2.3 (1.7–3.1) **	1.3 (0.9–1.9)	7.3(4.8–11.3) **	1.8 (1.1–2.8) *
Secondary	1.9 (1.4–2.5) **	1.3 (0.9–1.8)	3.5 (2.3–5.4) **	1.5 (1.0–2.4) *
Higher	**1**	**1**	**1**	**1**
**Occupation**				
Not working	1.0 (0.9–1.2)	1.0 (0.9–1.2)	0.9 (0.7–1.0)	0.9 (0.8–1.1)
Working	**1**	**1**	**1**	**1**
**Wealth Index**				
Poorest	2.9 (2.3–3.8) **	3.6 (2.6–5.1) **	14.6(10.7–20.1) **	10.3 (7.0–15.2) **
Poorer	2.3 (1.9–2.9) **	2.6 (1.9–3.5) **	7.3 (5.3–9.9) **	5.4 (3.7–7.8) **
Middle	1.7 (1.3–2.1) **	2.0 (1.5–2.8) **	4.1 (3.1–5.5) **	3.3 (2.3–4.8) **
Richer	1.5 (1.2–1.8) **	1.5 (1.2–1.9) *	2.4 (1.8–3.1) **	2.0 (1.4–2.8) **
Richest	**1**	**1**	**1**	**1**
**Husband’s occupation**				
White collar	0.7 (0.5–0.8) *	0.9 (0.7–1.3)	0.3 (0.2–0.5) **	0.9 (0.6 t- 1.3)
Blue collar	**1**	**1**	**1**	**1**
**Husband’s education**				
None	2.2 (1.5–3.5) **	1.1 (0.7–1.9)	11.1(6.6–18.6) **	1.6 (0.9–2.8)
Primary	2.7 (1.9–3.9) **	1.5 (1.0–2.3) *	7.5 (4.7–11.8) **	1.4 (0.8–2.3)
Secondary	2.1 (1.4–2.9) **	1.4 (0.9–2.1)	3.8 (2.4–6.1) **	1.2 (0.8–2.1)
Higher	**1**	**1**	**1**	**1**
**No of living children**				
No	**1**	**1**	**1**	**1**
1–3	1.0 (0.8–1.3)	0.9 (0.7–1.1)	1.2 (0.9–1.4)	1.0 (0.8–1.3)
> = 4	1.1 (0.9–1.4)	0.8 (0.6–1.1)	2.0 (1.6–2.5) **	1.1 (0.8–1.5)
**Family structure**				
Nuclear	**1**	**1**	**1**	**1**
Extended	0.8 (0.7 t- 0.9) *	1.0 (0.8–1.1)	0.6 (0.5–0.7) **	0.9 (0.8 t- 1.1)

*OR* Odds ratio, *AOR* Adjusted Odds ratio, *95%CI* 95% Confidence Interval

** *p* < 0.001, *p < 0.05

For the one-barrier group, rural residence was associated with a 1.3-times higher chance of experiencing barriers when compared to urban residence (95% CI: 1.0–1.8). Rural residence made it 1.8 times more likely that a woman would face more than one barrier when compared to urban residence (95% CI: 1.6–2.1). Women who had no education were more likely than educated women to have one barrier (AOR 1.8, 95% CI: 1.0–1.8). The lower a woman’s education level, the more likely she would be to face more barriers to accessing health care. As the wealth index increased, the risk of facing barriers decreased in both groups. Women from the poorest households were 10.3 more likely to face more than one barrier than the women from the richest households (95% CI: 7.0–15.2) and 3.6 times more likely to have one barrier (95% CI: 2.6–5.1). The odds of having one barrier was higher for women whose husbands had primary education (AOR 1.5, 95% CI: 1.0–2.3). Women with more children were 2.0 times more likely to face more than one barrier when compared to women with fewer children (95% CI:1.6–2.5).

## Discussion

This study was conducted to identify the association between empowerment among currently married women and the barriers to accessing health care based on MDHS (2015–16) data. About half of the women who participated faced barriers while attempting to access health care. In this study, barriers were evaluated by asking whether the respondent faced barriers when accessing health care in terms of getting permission to go, getting money to go, the distance to the health facility, and not wanting to go alone. These factors negatively influenced accessing health care in different settings [22–25]. A qualitative study done in a country in West Africa stated that poor health decision making and the unaffordability of health care were major barriers to accessing health care for women [26]. A study done in the ethnic minority regions of Northeastern Myanmar showed there was a gender-based inequality in health care access in those regions. Women were 45% less likely to seek inpatient treatment and 14% less likely to seek outpatient services than men [27]. Rural and ethnic minority women in Myanmar, in particular, could barely achieve equal rights with men.

After adjusting for some variables, it was found that women with a high empowerment score experienced fewer barriers. Women’s empowerment had a significant impact on accessing health care, confirming the results of previous studies [9, 23–25]. A study done by DHS data in Myanmar stated more than 80% of married women were participated in the decision-making process [28]. In Myanmar, due to cultural and social norms, women traditionally participate in domestic decisions. But women’s empowerment is context-specific, and many other aspects have to be considered. Myanmar was used to claim as high empowerment for women within the region due to cultural and religious beliefs, and most of the evidence was based on the economic aspect only. According to the economic forum 2020, Myanmar ranked 57 and scored 0.977 in the Global Gender Gap Index rankings by subindex [15]. In the report, the main measured health indicators were sex ratio at birth and healthy life expectancy. Therefore, the report could not cover the whole picture of the health care access of women in Myanmar. On the other hand, this study showed women in Myanmar still had problems accessing health care, and it was influenced by women’s empowerment, which was still neglected in Myanmar.

However, women’s empowerment was not the only variable with a significant association with barriers to accessing health care. Women from rural areas still faced more barriers compared to those from urban areas. It might be that geographical and transportation difficulties were one of the main causes of barriers to accessing health care in different states and regions of Myanmar. Moreover, unequal resource allocation caused disparities in health and health care in Myanmar. Conventional budget allocation, which is based on population and infrastructure, gave disproportionately more resources to more developed regions, urban areas, and places with better health and fewer resources to remote states with high health needs [29]. Together with geographical and transportation difficulties, other factors, including women’s empowerment, might influence the health-seeking behavior of women [21, 30]. In the study done in Bangladesh [11], access to health care for married women was better if they had higher education and married to educated men. These findings were consistent with those from a study done in Myanmar, where women who had a higher education participated more in decisions, including decisions about one’s own health care. Moreover, women married to educated men were more likely to participate in the decision-making process [28]. Therefore, respondents’ education and husbands’ education influenced the wife’s access to health care [5, 25]. In our study, we found that women who had more than four children were more likely to face barriers to accessing health care. A study done in Zambia stated the same, as the lack of family planning in certain families resulted in a woman having 2–3 children under the age of five at one time, therefore making it difficult for her to go with all of them to the health facility [31].

Since this study was a secondary data analysis, the variables were limited to women’s empowerment and barriers to accessing health care, along with influencing factors such as diversities in religion and ethnicity, relationships among household members, perceptions of health care access, and readiness of health care providers, which were not included in MDHS 2015–16. Women’s empowerment is a complex concept, and we could not include other factors, cultural contents, or social contents. Moreover, the findings of this study cannot be generalized to all women in Myanmar since only currently married women were included. Further qualitative studies should be considered to obtain more information to link women’s empowerment with health care, as well as other development sectors.

## Conclusion

The study investigated the association between women’s empowerment and barriers to accessing health care. Women’s empowerment was an important determinant of one’s ability to access health care, especially in rural areas. Women from rural areas experienced more barriers to accessing health care. Barriers to access to health care were reduced for women from rich households, who had attained higher education, who had educated husbands, and who had few children. We believe that the present findings would contribute to the policy formulation in reducing health inequity issues in terms of increasing women’s empowerment by enabling women getting equal rights to education and jobs.

## Supplementary Information

**Additional file 1.** Women’s empowerment indicator factor component after factor analysis.

## Data Availability

The datasets analyzed during the current study are available in the https://dhsprogram.com/data/dataset/Myanmar_Standard-DHS_2016.cfm?flag=0. This article is present on a university repository website and can be accessed on https://www.researchsquare.com/article/a28a9ef9-39d2-4785-b5c9-e631e2046e41/v1 . This article is not published nor is under publication elsewhere.

## Abbreviations

DHS: Demographic and Health Surveys
MDHS: Myanmar Demographic and Health Survey
USAID: United States Agency for International Development

## References

[1] Ministry of Health and Sports. Myanmar National Health Plan (2017-2021): The Republic of the Union of Myanmar; 2016. http://mohs.gov.mm/Main/content/publication/national-health-plan-2017-2021-eng

[2] Brennan E. Myanmar’s Public Health system and policy: Improving but inequality still looms large: Tea Circle; 2017. https://teacircleoxford.com/2017/08/30/myanmars-public-health-system-and-policy-improving-but-inequality-still-looms-large-2/ (Accessed 10 June 2019)

[3] Han SM, Rahman MM, Rahman MS (2018). Progress towards universal health coverage in Myanmar: a national and subnational assessment. Lancet Glob Health.

[4] Women’s Empowerment in Reproductive Decision-making Needs Attention among Iranian Women. https://www.ncbi.nlm.nih.gov/pmc/articles/PMC5971191/ (Accessed 18 June 2020).

[5] Correlation Between Social Determinants of Health and Women’s Empowerment in Reproductive Decision-Making Among Iranian Women - PubMed. https://pubmed.ncbi.nlm.nih.gov/27157184/ (Accessed 18 June 2020). 10.5539/gjhs.v8n9p312 PMC5064082 27157184

[6] Kishor S, Subaiya L (2008). Understanding womens empowerment: a comparative analysis of demographic and health surveys (DHS) data.

[7] Interaction eldis. Barriers to access of health services. http://interactions.eldis.org/urbanisation-and-health/policy-findings/barriers-access-health-services (Accessed 10 Aug 2019).

[8] Pratley P (2016). Associations between quantitative measures of Women’s Empowerment and access to care and health status for mothers and their children: a systematic review of evidence from the developing world. Soc Sci Med.

[9] Mainuddin A, Begum HA, Rawal LB (2015). Women Empowerment and its relation with health seeking behavior in Bangladesh. J Family Reprod Health.

[10] Women’s Empowerment and Contraceptive Use: The role of independent versus couples’ decision-making, From a Lower Middle Income Country Perspective - PubMed. https://pubmed.ncbi.nlm.nih.gov/25119727/ (Accessed 18 June 2020). 10.1371/journal.pone.0104633 PMC4131908 25119727

[11] Hasan MN, Uddin MSG (2016). Women Empowerment through health seeking behavior in Bangladesh: evidence from a National Survey. South East Asia J Public Health.

[12] Morgan SP, Niraula BB (1995). Gender inequality and fertility in two Nepali villages. Popul Dev Rev.

[13] Hindin MJ (2000). Women’s autonomy, women’s status and fertility-related behavior in Zimbabwe. Popul Res Policy Rev.

[14] Upadhyay UD, Hindin MJ (2005). Do higher status and more autonomous women have longer birth intervals? Results from Cebu, Philippines. Soc Sci Med.

[15] Global Gender Gap Report 2020 - Reports - World Economic Forum. http://reports.weforum.org/global-gender-gap-report-2020/the-global-gender-gap-index-2020/results-and-analysis/ (Accessed 17 Sept 2020).

[16] Department of Population. The 2014 Myanmar population and housing census: the Union report: The Republic of the Union of Myanmar. Naypyitaw: Ministry of Immigration and Population; 2015.

[17] United Nations Fund for Population Activities. Report on Situation Analysis of Population and Development, Reproductive Health, and Gender in Myanmar. Yangon: UNFPA; 2010.

[18] Kachin women’s peace network, Gender equality network (2013). Women’s Needs Assessment IDP Camps, Kachin State.

[19] Ministry of Health and Sports (MoHS) and ICF (2017). Myanmar Deographic and Health Survey (2015–16).

[20] Saw YM, Than TM, Thaung Y (2019). Myanmar’s human resources for health: current situation and its challenges. Heliyon.

[21] DHS program. Guide to DHS Statistics. https://dhsprogram.com/Data/Guide-to-DHS-Statistics/index.cfm (Accessed 19 Sept 2020).

[22] ICF, Ministry of Health and Sports (MoHS) (2017). Myanmar Demographic and Health Survey 2015–16.

[23] Bohren MA, Hunter EC, Munthe-Kaas HM (2014). Facilitators and barriers to facility-based delivery in low- and middle-income countries: a qualitative evidence synthesis. Reprod Health.

[24] Moyer CA, Mustafa A (2013). Drivers and deterrents of facility delivery in sub-Saharan Africa: a systematic review. Reprod Health.

[25] Gabrysch S, Campbell OMR. Still too far to walk: literature review of the determinants of delivery service use. BMC Pregnancy Childbirth. 2009;9. 10.1186/1471-2393-9-34. 10.1186/1471-2393-9-34 PMC2744662 19671156

[26] Essendi H, Mills S, Fotso J-C (2011). Barriers to formal emergency obstetric care services’ utilization. J Urban Health.

[27] Tang K, Zhao Y, Li B (2017). Health inequity on access to services in the ethnic minority regions of northeastern Myanmar: a cross-sectional study. BMJ Open.

[28] The DHS Program - Women’s Empowerment in Myanmar: An Analysis of DHS Data for Married Women Age 15–49 (English). https://dhsprogram.com/publications/publication-wp143-working-papers.cfm (Accessed 12 June 2020).

[29] Zaw PPT, Htoo TS, Pham NM (2015). Disparities in health and health care in Myanmar. Lancet.

[30] MEASURE DHS/ICF International (2013). Standared Recode Manual for DHS 6.

[31] Factors Perceived by Caretakers as Barriers to Health Care for Under-Five Children in Mazabuka District, Zambia. https://www.hindawi.com/journals/isrn/2013/905836/ (Accessed 17 Sept 2020).

