# Supplementary material for: Empowerment and health care access barriers among currently married women in Myanmar
Source: BMC Public Health. 2021 Jan 15;21:139. doi: 10.1186/s12889-021-10181-5 (PMC7809752; doi:10.1186/s12889-021-10181-5)
Supplement: Supplementary file 1 — Additional file 1. Women’s empowerment indicator factor component after factor analysis. [file 12889_2021_10181_MOESM1_ESM.docx]

**Appendix. Women's empowerment indicator factor component after factor analysis**

| **Variables** | **Factor1**  (Accounted for 89% of total variance) |
| --- | --- |
| Decision on large household purchase | -0.0067 |
| Decision on visits to family and relatives | 0.0179 |
| Decision on what to do with money husband earned | -0.0010 |
| **Beating justified if wife goes out without telling husband** | **0.6168** |
| **Beating justified if wife neglects the children** | **0.6154** |
| Beating justified if wife argues with husband | 0.2927 |
| Beating justified if wife refuses to have sex | 0.2731 |
| Beating justified if wife burns the food | 0.3537 |

Component factors are shown after the application of oblique rotation: components are bold if they are +/- 0.4
